# Local Excision Inadequate in the Treatment of Anal Canal Leiomyosarcoma

**DOI:** 10.4103/1319-3767.65192

**Published:** 2010-07

**Authors:** G. Krishna Kumar, Sarath S. Chandra, R. Krishnan

**Affiliations:** Department of General Surgery, Jawaharlal Institute of Postgraduate Medical Education and Research, Pondicherry, India; 1 Department of Pathology, Jawaharlal Institute of Postgraduate Medical Education and Research, Pondicherry, India

**Keywords:** Anal canal, leiomyosarcoma, local excision

## Abstract

Leiomyosarcoma of the anal canal is an uncommon neoplasm of the gastrointestinal tract. We report a 45-year-old lady with anal canal leiomyosarcoma. In view of its rarity, we report its presentation and management. In the setting of a recurrent tumor with high-grade histological appearance, local excision would be deemed unsafe.

Leiomyosarcoma is the most common non-epithelial gastrointestinal malignancy, seen generally in the fifth/ sixth decade of life. It occurs most frequently in the stomach, followed by small bowel and colon. It may arise from the smooth muscle of muscularis propria, muscularis mucosa or the blood vessel wall. In addition, arrectores pilorum muscle of the subcutis may be another site of origin, especially in the anal canal.[1]

We report a case of recurrent leiomyosarcoma of the anal canal. The case is being reported for its rarity of occurrence.

## CASE REPORT

A 45-year-old lady presented with history of mass descending per rectum and occasional bleeding per rectum of 10 months’ duration. No definite history of constipation or alteration in bowel habits was present. She had consulted a surgeon 6 months ago, with similar complaints, who had diagnosed spindle cell tumor of anal canal based on a cytology report. In view of the ‘apparent benign appearance,’ local excision was done by the surgeon, but the tumor recurred within a month. At this point, she was referred to us.

She was of average build and nourishment. General systemic examination was unremarkable. Per abdomen, there was no organomegaly or mass demonstrable. On rectal examination, there was a 5 × 4 cm polypoid, mobile mass confined to the posterior wall, about 2 cm from the anal verge. There was no induration of the mucosa around the base of the mass.

Fine-needle aspiration cytology of the mass revealed a spindle cell tumor with high mitotic activity, probably leiomyosarcoma. Chest X-ray and ultrasound abdomen did not reveal any visceral metastases. Contrast CT scan revealed the tumor to be confined to the anal canal, without metastases or spread to the adjacent viscera. Hence she was planned for an abdomino-perineal resection.

**Figure 1:**
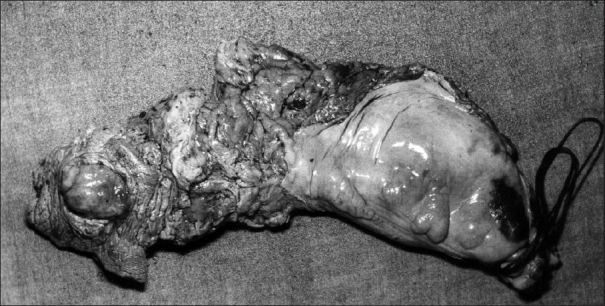
Excised specimen showing leiomyosarcoma in the anal canal

**Figure 2:**
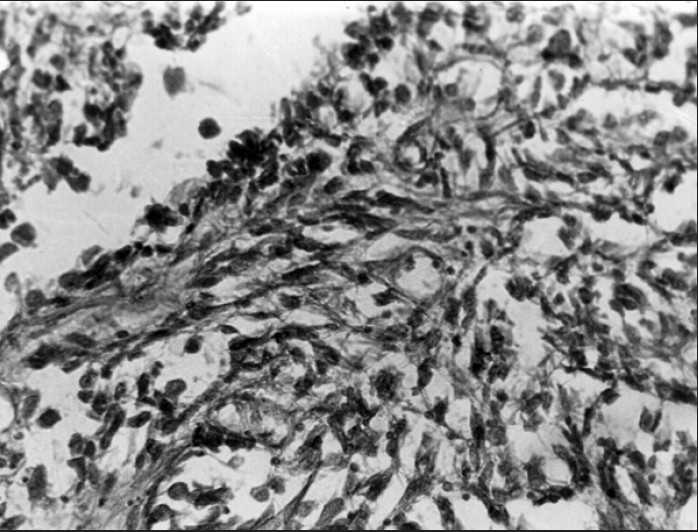
Predominantly spindle cell tumor with epithelioid appearance (x100)

Intraoperatively, the tumor was found to be confined to the anal canal, without nodal/ visceral/ peritoneal deposits. She had an uneventful post-op course. Grossly [Figure 1] the resected specimen showed an intra-luminal polypoidal mass, 4 × 3 cm in size, extending from just above the anal verge up to the dentate line. There was no serosal infiltration/ lymph nodal involvement. Histopathological examination [Figure 2] revealed a spindle cell tumor with epithelioid appearance, infiltrating the muscle coat. The tumor showed a high mitotic activity (> 1 mitosis / 10 high-power field). Stains for melanin were negative. At the 2-year follow-up, the patient was doing well with a healthy stoma. No recurrence was detected on clinical examination and imaging.

## DISCUSSION

Stromal tumors of the rectum and anal canal are rare, representing 0.02% to 0.03% of malignant neoplasms in the region. Even in the large intestine, they are unusual neoplasms, comprising less than 0.1% of all malignancies of the colon and rectum. Current advice in their management is treatment by abdomino-perineal resection.[2] Local excision, preserving the anal sphincter, using a posterior parasacral approach has been reported.[3]

There has been an apparent increase reported in females. Palpable mass, hemorrhage and pain/ discomfort were the most common symptoms reported at presentation. With regard to size, majority of the leiomyosarcomas measured 5-9 cm at diagnosis.[2]

Age at presentation varied from 44 to 76 years (median, 63 years) in the series of 8 patients with isolated anorectal leiomyosarcoma reported by Tjandra *et al*.[4] All patients were symptomatic at presentation. All tumors involved the muscularis propria of the low and/ or mid rectum, with three tumors also involving the anal sphincters. The tumor size ranged from 1.2 to 10 cm. Mucosal involvement occurred in only three patients, and there was no lymph node involvement. All showed microscopic infiltration at the advancing border despite macroscopic circumscription. Only one patient was thought to have a tumor sufficiently small (3 cm) and localized on clinical and intrarectal ultrasound examination, to be suitable for wide local excision; this patient remained tumor free after 2 years. The remaining patients (88%) were treated by abdomino-perineal resection. The disease-free interval in this latter group ranged from 3 months to 4.5 years. All recurrences were detected within 15 months of surgery, and the mean interval from detection of recurrence to death was 9 months. Using a histological grading system that included tumor differentiation, mitotic count and amount of necrosis, high-grade sarcomas were associated with a worse prognosis. Other factors associated with a poor outcome included large tumor size (>6-7 cm) and prior incomplete local excision.[4]

In our patient, abdomino-perineal resection was done in view of recurrence after local excision and the cytological appearance of high-grade leiomyosarcoma. Limited local excision of small tumors without high-grade cytology appears justified if sufficiently wide, since malignancy of leiomyosarcoma appears to remain circumscribed over long periods. However, this limited procedure runs the risk of local recurrence, with the need for abdomino-perineal resection later.[5] Local excision of anal canal leiomyosarcoma is inadequate, especially in the setting of a large tumor (>4 cm) with high mitotic activity (>1 mitosis/10 high-power field). Hence abdomino-perineal resection is a safer procedure to avoid recurrence and also the accepted mode of treatment in such cases with poor prognostic factors.
